# Transmit BeampattarnOptimization for Automotive MIMO Radar Coexistedwith Communication in V2V Networks

**DOI:** 10.3390/s20041100

**Published:** 2020-02-17

**Authors:** Zheng-Ming Jiang, Pei-Chang Zhang, Lei Huang, Xin He, Ji-Hong Zhang, Mohamed Rihan

**Affiliations:** 1 Guangdong Laboratory of Artificial-Intelligence and Cyber-Economics (SZ), Shenzhen University, Shenzhen 518060, Chinalhuang@szu.edu.cn (L.H.); hexin@szu.edu.cn (X.H.); zhangjh@szu.edu.cn (J.-H.Z.); 2 School of Mathematics and Statistics, Shenzhen University, Shenzhen 518060, China; mohamed.elmelegy@szu.edu.cn; 3 Electronics and Electrical Communications Engineering, Faculty of Electronic Engineering, Menoufia University, Menouf 32952, Egypt

**Keywords:** spectrum sharing, coexisting radar and communication, block coordinate descent, vehicle-to-vehicle (V2V), the ellipsoid sub-gradient method

## Abstract

Due to the flourishing development of vehicle-to-vehicle (V2V) communications and autonomous driving, interference between radar sensing and communication signals becomes a challenging issue. We propose a transmit beamforming based spectrum sharing scheme to achieve peaceful coexistence between automotive multiple-input multiple-out (MIMO) radar and communication systems. Our objective is to maximize the signal-to-interference-plus-noise ratio (SINR) of the automotive radar receiver subject to the communication capacity and the transmitted power budget constraints to optimize both the communication covariance matrix and the radar transmit precoder. The formulated optimization problem is non-convex, which is converted to convex by introducing a new slack variable and then solving it using the block coordinate descent, also called alternation optimization, approach. Additionally, the ellipsoid sub-gradient method is then employed to reduce the computational complexity. Simulation results demonstrate that our proposed scheme outperforms the conventional schemes.

## 1. Introduction

With the development of the fifth-generation (5G) mobile communication networks, future communication systems can support various types of unmanned devices, and accordingly, more research efforts should be made in the fields related to vehicle-to-vehicle (V2V) communications and autonomous driving capabilities. However, the explosive increase of communication devices and mobile traffic causes the spectrum assigned to the communication sector to be extremely congested. On the other hand, it is noted that the spectrum bands assigned to radar are always underutilized [1,2]. Accordingly, it was not surprising to find some calls to make both the radar and communication systems share the same spectral resources [3,4,5,6].

The coexistence of radar and communication systems that share the same spectrum resources may lead to the origination of mutual electromagnetic interference between the two systems. To eliminate this interference, A.Khawar proposed a null-space projection (NSP) approach [7,8]. The main idea behind NSP is to project the radar signal on the null-space of the interference channels between the radar and the communication base station (BS), in order to eliminate inter-system interference. The works in [3,4,5,6] proposed the employment of different optimization techniques to eliminate the interference between the two systems. Specifically, in [3], under the constraints of channel capacity and total power budget, the covariance matrix of the communication signal and precoding matrix of radar were jointly optimized by maximizing the signal-to-interference plus noise (SINR) ratio of the radar system. Similar works are described in peer-to-peer (P2P) and multiple-input multiple-out (MIMO) communication systems that coexist with MIMO radar based on the concepts of matrix completion [4,5,6].

The interference problem in automotive radar scenarios has been addressed in many studies [9,10,11,12,13,14,15]. In [9], the authors addressed interference mitigation in automotive radars using pseudo-random cyclic orthogonal sequences. They studied the impact of radar waveform design and processing required at the radar receiver on the radar-to-radar interference and the effect of such a kind of interference on the sensing performance. In [10], different methods that were proposed to mitigate interference between many radar sensors in car-to-car sensing applications were summarized. Additionally, the work in [11] also proposed an approach for mutual interference mitigation between automotive radars based on frequency modulated continuous wave chirp diversity. The author of [12] presented a radar communications approach that was able to reduce the mutual interference between vehicles while offering a communication functionality in addition to the sensing function. Furthermore, the performance comparison of different mutual automotive radar interference mitigation algorithms was addressed in [13]. Furthermore, a mathematical investigation for mutual interference between automotive FMCW radar sensors was presented in [14], and interference generated due to the existence of many millimeter-wave radar systems was studied in [15]. However, none of these studies addressed the interference between automotive radars and V2V communications, so our work comes to fill this gap.

In this paper, we consider the case where a recipient vehicle equipped with collocated MIMO radar transmits the signal to detect surrounding obstacles, while receiving communication signals from other vehicles. This scenario will be widely present in future autonomous driving based vehicular networks. Specifically, this article proposes a new spectrum sharing scheme to achieve peaceful coexistence between automotive MIMO radar and V2V communication signals. The main contributions in our work can be summarized as follows:

1. Firstly, we propose a transmitter beampattern based spectrum sharing scheme to achieve peaceful coexistence between automotive MIMO radar and V2V communications. The objective of this scheme is to find the optimal radar transmit precoding matrix and communication signal covariance matrix through maximizing the SINR of the radar receiver subject to the communication sum-rate/capacity and transmitted power budget constraints.
2. The formulated optimization problem turns out to be non-convex due to the non-convexity of the communication capacity constraint. In order to circumvent this issue, a slack variable is added to convert the problem into a convex one, and were use the block coordinate descent, also known as alternation optimization, method to solve this problem.
3. Furthermore, in order to reduce the computational complexity of the proposed scheme, the ellipsoid sub-gradient process is employed.

The remaining part of this article is organized as follows. The system model is described in Section 2. The proposed approach is introduced in Section 3. Simulation results are provided in Section 4. We conclude the paper in Section 5.

## 2. System Model

**Figure 1:**
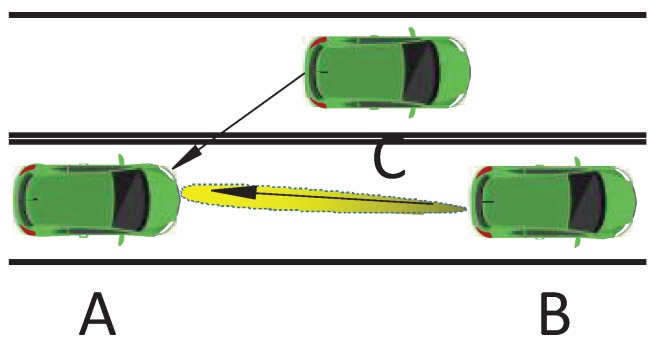
Coexistence scenario between automotive MIMO radar and V2V communication.

In this work, we consider a vehicular communication network that consists of three vehicles. Without loss of generality, we also consider that the vehicles are running within the same velocity range and within the same street as shown in Figure 1, and the mobility issues are taken into account through creating the communication channels with the Doppler effect included. Figure 1 shows the considered scenario of the V2V communication network, where each vehicle contains an automotive millimeter-wave MIMO radar and a communication system for exchanging messages between vehicles. Specifically, our scenario considers a recipient vehicle A, a source vehicle B, and the target vehicle C. The vehicle A sends the radar signal to sense the surrounding vehicles, while simultaneously receiving the signal from the source vehicle B. The vehicles A and B are equipped with *N* and *M* antennas arranged into uniform linear arrays (ULA) with the inter-element spacing equal to λ/2. We assume that the radar system shares the same carrier frequency with the communication system, in the sub 6 GHz band. Thus, mutual interference exists between the radar system and the communication system.

The scenario in abstract form consists of a transmitter, receiver, and a channel. The transceivers in VANET or V2V communications are dual function radar and communication transceivers, which can employ the signals to provide both sensing and communication functionalities. Therefore, whether the vehicle is a transmitter or receiver, the only change will be in the channel. If we assume a symmetrical channel, changing the role of the vehicles in the scenario from transmitter to receiver and vice versa will not change the mathematical analysis greatly, and the duality theorem can be used to evaluate the precoding matrices in different spatial arrangements [16]. Furthermore, in order to consider the relative motion between different vehicles, we include the Doppler frequency shifts within the created channels. Without loss of generality, we assume only a three vehicle scenario as proof of concept, which can be easily extended to the general case of a larger number of vehicles.

Suppose that both the automotive radar and communication systems use narrow-band waveforms with the same symbol rate and are synchronized in terms of sampling times. Then, the signal received by the vehicle A during an *L*-symbol duration may be expressed as:

(1) $$\begin{array}{c} Y=APS+HX+Q, \end{array}$$

where P is the transmit precoding matrix of the radar. The matrix S=[s(1),⋯,s(l),⋯,s(L)], with $s\left(l\right)={\left[s_{1}\left(l\right),\cdots ,s_{N}\left(l\right)\right]}^{T}$ being the $l^{\mathrm{th}}$ snapshot across the transmitted antennas. We assume that the transmitted waveforms are orthogonal, i.e., it holds that $SS^{H}=I$. The matrix X=[x(1),⋯,x(l),⋯,x(L)] with $x\left(l\right)\in C^{M\times 1}$ being the transmitted vector by the communication transmit antennas during the $l^{\mathrm{th}}$ symbol duration. The matrix Q is additive, while Gaussian noise with distributed $\mathrm{CN}(0,\sigma^{2})$, where σ is the variance of the Gaussian distribution. We assume that the channel state information (CSI) from B to A is known by vehicle A. The communication channel between vehicle B and vehicle A is denoted as $H\in C^{N\times M}$, which is circularly symmetric complex Gaussian CN(0,1). We assume that *K* targets with distinct reflection coefficients $\alpha_{k}$ with k∈{0,⋯,K}, Doppler shifts $f_{k}$, and angles $\theta_{k}$ fall in the same range bin. Therefore, based on the clutter-free model of [17,18], the target response matrix $A\in C^{N\times N}$ may be described as:

(2) $$\begin{array}{c} A=V_{r}\Theta V_{t}^{T}, \end{array}$$

where $V_{r}=\left[v_{r}\left(\theta_{1}\right),\cdots ,v_{r}\left(\theta_{K}\right)\right]$ and $V_{t}=\left[v_{t}\left(\theta_{1}\right),\cdots ,v_{t}\left(\theta_{K}\right)\right]$ are the receive and transmit steering matrices of the *K* targets, respectively. The vector $v_{t}\left(\theta_{k}\right)\in C^{N\times 1}$ corresponds to the transmit steering vector, which is defined as:

(3) $$\begin{array}{c} v_{t}\left(\theta_{k}\right)={\left[e^{-j\pi 0\sin(\theta_{k})},\cdots ,e^{-j\pi \left(N-1\right)\sin\left(\theta_{k}\right)}\right]}^{T}, \end{array}$$

and the receiver steering vector $v_{r}\left(\theta_{k}\right)\in^{N\times 1}$ can be defined in the same way as in (3) based on the parameter of the receiver array. The Doppler shift matrix $\Theta =\mathrm{diag}(\left[\alpha_{1}e^{j2\pi f_{1}},\cdots ,\alpha_{K}e^{j2\pi f_{K}}\right])$. In our signal model, the signal of one pulse period received by the radar is sampled with a signal with a very small pulse width. Therefore, the Doppler shift can be considered as constant and be absorbed into the target RCS [19,20]. Based on the above assumptions, the corresponding received SINR at vehicle A can be expressed as:

(4) $$\begin{array}{c} {\mathrm{SIN}R}_{r}=\frac{\mathrm{Tr}(A\Omega A^{H})}{\mathrm{Tr}\left(HR_{x}H^{H}\right)+\sigma^{2}}, \end{array}$$

where $R_{x}=XX^{H}$ is the communication signal covariance matrix and $\Omega =PP^{H}$. In order to evaluate the performance of the communication receiver, it is found that it is very difficult to determine the instantaneous information rate when the radar interference plus noise rate (INR) is not Gaussian [21]. Instead, we relax the problem by evaluating the lower bound of the capacity. According to [21], the low bound for the rate per channel use and per degree of freedom (DoF) can be achieved when the distribution of the communication code-word X follows a Gaussian $\mathrm{CN}(0,R_{x})$. This can be mathematically expressed as [21]:

(5) $$\begin{array}{c} C\left(R_{x},\Omega \right)=\log_{2}\left|I+P_{\mathrm{inc}}^{-1}HR_{x}H^{H}\right|, \end{array}$$

where $P_{inc}=A\Omega A^{H}+\sigma^{H}I$ is the interference plus noise matrix at the communication receiver. The constraints on the transmitted power budget of the radar and V2V communication are given by:

(6) $$\begin{array}{c} \mathrm{Tr}\left(R_{x}\right)\leq P_{c}, \end{array}$$

and:

(7) $$\begin{array}{c} \mathrm{Tr}\left({\mathrm{PP}}^{H}\right)=\mathrm{Tr}\left(\Omega \right)\leq P_{r}, \end{array}$$

respectively. The parameter $P_{c}$ is the maximum transmitted power of the communication transmitter, and $P_{r}$ corresponds to the maximum transmitted power of the radar transmitter.

## 3. The Proposed Approach

The objective of this work is to maximize the SINR of the radar receiver subject to the communication sum-rate/capacity and the transmitted power constraints, which may be described as:

(8) $$\begin{array}{cc} \; & \max_{R_{x},\Omega }\;\;{\mathrm{SINR}}_{r}\\ s.t.\;\;\;\; & C(R_{x},\Omega )\geq C,\\ \; & \mathrm{Tr}\left(R_{x}\right)\leq P_{c},\\ \; & \mathrm{Tr}\left(\Omega \right)\leq P_{r}. \end{array}$$

where *C* is the communication sum-rate threshold, which is considered as a measure of the quality-of-service (QoS). However, the optimization problem in (13) is non-convex because of the non-convexity of the communication capacity constraint. In order to circumvent these issues, we develop an alternation optimization method to solve this problem. In particular, we first solve for $R_{x}$ when Ω is fixed and then find out the optimum Ω given the fixed $R_{x}$. This process will be repeated until the optimal objective function converges. In the rest of this section, we will describe the optimization with respect to $R_{x}$ with Ω and with respect to Ω for given $R_{x}$, respectively.

### 3.1. The Alternating Iteration with Respect to $R_{x}$

In this subsection, we first need to obtain the covariance matrix $R_{x}$ of the communication system while keeping Ω constant. After some mathematical manipulations, the problem (13) can be rewritten as:

(9) $$\begin{array}{cc} \; & \min_{R_{x}}\;\;\mathrm{Tr}\left(HR_{x}H^{H}\right)\\ s.t.\;\;\; & \log_{2}\left|I+P_{\mathrm{inc}}^{-1}HR_{x}H^{H}\right|\geq C,\\ \; & \mathrm{Tr}\left(R_{x}\right)\leq P_{c}, \end{array}$$

The optimization problem in (9) is now convex. The interior-point method can be used to solve this problem [22]. However, the computational complexity of the interior-point method is $O({\left({\left(N\right)}^{2}\right)}^{3.5})$. To handle the high computational complexity problem, the problem can be solved by using the Lagrangian dual method. The Lagrangian function of (9) can be given by:

(10) $$\begin{array}{cc} \; & L=\mathrm{Tr}\left(HR_{x}R^{H}\right)+\lambda_{1}(C-\log_{2}|I+P_{inc}^{-1}HR_{x}H^{H}\left|\right)+\lambda_{2}\left(\mathrm{Tr}\left(R_{x}\right)-P_{c}\right), \end{array}$$

where $\lambda_{1}$ and $\lambda_{2}$ are the dual variables related to the communication capacity and the power of communication transmitter constraints. Then, the dual problem of (9) becomes:

(11) $$\begin{array}{c} \max_{\lambda_{1},\lambda_{2}⪰0}g\left(\lambda_{1},\lambda_{2}\right), \end{array}$$

where the dual function $g(\lambda_{1},\lambda_{2})$ is defined as:

(12) $$\begin{array}{c} g\left(\lambda_{1},\lambda_{2}\right)=\underset{R_{x}}{\inf}L\left(R_{x},\lambda_{1},\lambda_{2}\right). \end{array}$$

Since the problem (9) is convex, this means that the duality gap between the Lagrange dual problem and the problem (9) equals zero, and a strong duality holds. Thus, the optimal solution of the problem (9) equals that of the problem (10). In order to obtain the solution of the problem (11), an iterative algorithm can be exploited. We first obtain the optimal $R_{x}$ with given $\lambda^{T}=\left[\lambda_{1},\lambda_{1}\right]$. According to Appendix A, the optimal covariance matrix $R_{x}$ with constant λ can be expressed as:

(13) $$\begin{array}{c} R_{x}^{*}\left(\lambda_{1},\lambda_{2}\right)=T^{-\frac{1}{2}}U\Sigma U^{H}T^{-\frac{1}{2}}, \end{array}$$

where we assume that $T=({\mathrm{HH}}^{H}+\lambda_{2}I)$ and U is the right singular matrix of $\tilde{H}=P_{inc}^{-\frac{1}{2}}HT^{-\frac{1}{2}}$. Substituting the value of $R_{x}^{*}\left(\lambda_{1},\lambda_{2}\right)$ from (13) into the Lagrangian function, we can obtain the dual function $g(\lambda_{1},\lambda_{2})$, then the optimal problem becomes equivalent to the optimal solution of (11). In order to find out the optimal dual variables $\lambda_{1}^{*}$ and $\lambda_{2}^{*}$, the sub-gradient based ellipsoid method is adopted to update λ.

Before introducing the sub-gradient based ellipsoid method, let us first state some basic knowledge about ellipsoid. Algebraically, we can represent the ellipsoid $E_{k}$ as:

(14) $$\begin{array}{c} E_{k}=\left\{v\in R^{n}|{\left(v-\lambda \right)}^{T}Z^{-1}\left(v-\lambda \right)\leq 1\right\}, \end{array}$$

where Z is a positive definite symmetric matrix, which determines the shape of the ellipsoid and λ is the center of the ellipsoid. The sub-gradient $d_{\lambda }={\left[d_{\lambda_{1}},d_{\lambda_{2}}\right]}^{T}$ of $g(\lambda_{1},\lambda_{2})$ at the center λ of the ellipsoid can be expressed as:

(15) $$\begin{array}{cc} \; & d_{\lambda_{1}}=\mathrm{Tr}\left(R_{x}^{*}\right)-P_{c},\\ \; & d_{\lambda_{2}}=C-\log_{2}\left(I+P_{\mathrm{inc}}^{-1}HR_{x}^{*}R^{H}\right). \end{array}$$

Then, the normalized sub-gradient is given by:

(16) $$\begin{array}{c} \hat{d}=\frac{1}{\sqrt{d_{\lambda }^{T}Z^{m}d_{\lambda }}}d_{\lambda }. \end{array}$$

The ellipsoid method constructs a sequence of ellipsoids $E_{0},\cdots ,E_{m},\cdots ,E_{Last_{i}t}$. We assume that the initial ellipsoid $E_{0}$ is large enough to include all feasible solutions, and within each subsequent iteration, we check whether $\sqrt{d_{\lambda }^{T}Z^{m}d_{\lambda }}<\epsilon$, where ϵ is a small positive constant. If it is correct, the ellipsoid iteration stops, and if it is not correct, we need to construct an ellipsoid that covers the previous feasible ellipsoid intersected with the feasible half-space. The relationship between the $\lambda_{m}$ and $Z_{m}$ of the $E_{m}$ and that of $E_{m+1}$ can be described as:

(17) $$\begin{array}{cc} \; & \lambda^{m+1}=\lambda^{m}-\frac{1}{n+1}Z^{m}\hat{d},\\ \; & Z^{m+1}=\frac{n^{2}}{n^{2}-1}\left(Z^{k}-\frac{2}{n+1}Z^{m}\hat{d}{\hat{d}}^{T}Z^{m}\right), \end{array}$$

where *n* is the number of variables. In this paper, *n* equals two. As was discussed above, the complexity of the algorithm solved (9) by the interior-point method is high. The Lagrange dual-decomposition method just needs to update the unknown $\lambda_{1}$ and $\lambda_{2}$. The update cost for the Lagrange dual-decomposition method is O(1) [23]. Therefore, it can reduce the computational complexity. The algorithm to obtain the optimal value of $R_{x}^{*}$ can be summarized as shown in Algorithm 1.
**Algorithm 1:** The sub-gradient based ellipsoid method.**Input**: $H,A,\sigma^{2},C,P_{r},\epsilon ,Z^{m}=\alpha I,\alpha \gg 1,\lambda_{1}^{m}\geq 0,\lambda_{2}^{m}\geq 0$. $\mathrm{Initialization}:m=0,\Omega =P_{r}/NI$. Repeat    1. Compute $R_{x}^{*}\left(\lambda_{1}^{m},\lambda_{2}^{m}\right)$ according to (13) with          fixing $\lambda_{1}^{m}$ and $\lambda_{2}^{m}$;     2. Calculate the sub-gradient $d_{\lambda }$ with (15);     3. Compute $Z^{m+1}$ and $\lambda^{m+1}$ according to (17);     4. m=m+1; **until**
$\sqrt{d_{\lambda }^{T}Z^{m}d_{\lambda }}<\epsilon$. **Output**: $R_{x}$.

The convergence and complexity analysis of the general ellipsoid sub-gradient algorithm was given in [24].

### 3.2. The Alternating Iteration with Respect to Ω

After some mathematical manipulation, the precoding matrix design problem for given $R_{x}$ can be expressed as:

(18) $$\begin{array}{cc} \; & \max_{\Omega }\;\;\mathrm{Tr}\left(A\Omega A^{H}\right)\\ s.t.\;\;\; & \log_{2}\left|I+P_{\mathrm{inc}}^{-1}HR_{x}H^{H}\right|\geq C,\\ \; & \mathrm{Tr}\left(\Omega \right)\leq P_{r}. \end{array}$$

It is very hard to solve the problem (18) due to the non-convexity of the constraint. To circumvent this issue, we transform the sum-rate maximization problem into another equivalent convex optimization problem.

**Lemma 1:**  *For any positive definite matrix $W\in C^{N\times N}$, we have:*

(19) $$\begin{array}{c} \log_{2}\left(\right|W^{-1}\left|\right)=\max_{\Phi }-\mathrm{Tr}(W\Phi )+\log_{2}\left|\Phi \right|+N, \end{array}$$

*and the optimal solution to the right-hand side of (19) is $\Phi =W^{-1}$.*

**Proof:**  We assume the function $f\left(\Phi \right)=\max_{\Phi }-\mathrm{Tr}(W\Phi )+\log_{2}\left|\Phi \right|+N$. Because f(Φ) is concave, therefore the optimal solution of the f(Φ) can be obtained when $\frac{\partial f(\Phi )}{\partial f\Phi }=0$ [25,26]. □

Using Lemma 1 and some mathematical manipulations, we can obtain:

(20) $$\begin{array}{cc} \; & \log_{2}|I+P_{inc}^{-1}HR_{x}H^{H}|=\log_{2}|G^{-1}A\Omega A^{H}+I|+\log_{2}\left|G\Phi \right|-\mathrm{Tr}\left(A^{H}\Phi A\Omega \right)-\mathrm{Tr}\left(\sigma^{2}\Phi \right)+N, \end{array}$$

where $G=\sigma^{H}I+HR_{x}H^{H}$. Appendix B gives a specific derivation of (20). Substituting (20) into (18), the optimal problem becomes:

(21) $$\begin{array}{cc} \; & \max_{\Omega }\;\;\mathrm{Tr}\left(A\Omega A^{H}\right)\\ s.t.\;\;\; & \log_{2}\left|G^{-1}A\Omega A^{H}+I\right|-\mathrm{Tr}\left(A^{H}\Phi A\Omega \right)\geq \bar{C},\\ \; & \mathrm{Tr}\left(\Omega \right)\leq P_{r}, \end{array}$$

where $\bar{C}=C-\log_{2}\left(|G\Phi |\right)+\mathrm{Tr}\left(\sigma^{2}\Phi \right)-N$. To reduce the computational complexity, the Lagrangian dual method can be also adapted to solve the problem (21). Then, the optimization problem with respect to Ω becomes:

(22) $$\begin{array}{c} \max_{\beta_{1},\beta_{2}\geq 0}\underset{\Omega }{\inf}L\left(\Omega ,\beta_{1},\beta_{2}\right), \end{array}$$

where $L=\mathrm{Tr}\left(\left(\beta_{1}A^{H}\Phi A-A^{H}A+\beta_{2}I\right)\Omega \right)+\beta_{1}(\bar{C}-\log_{2}|G^{-1}A\Omega A^{H}+I\left|\right)-\beta_{2}P_{r}$. The problem in (22) is similar to the problem in (A1). We can use the same method to solve the problem (22). The specific derivation process can be referenced to the solution process of $R_{x}$.

In order to analyze the convergence of iteration, we assume that the iterative alternation routine is $\cdots ,R_{x}^{j}\to \Omega^{j}\to R_{x}^{j+1}\to \Omega^{j+1},\cdots$. Since the optimization with respect to $R_{x}^{j}$ is a convex problem, we can obtain:

(23) $$\begin{array}{c} {\mathrm{SINR}}_{r}\left(R_{x}^{j},\Omega^{j}\right)\leq {\mathrm{SINR}}_{r}\left(R_{x}^{j+1},\Omega^{j}\right). \end{array}$$

At the same time, $\Omega^{j+1}$ is the optimum solution of (9), whereby we have:

(24) $$\begin{array}{c} {\mathrm{SINR}}_{r}\left(R_{x}^{j+1},\Omega^{j}\right)\leq {\mathrm{SINR}}_{r}\left(R_{x}^{j+1},\Omega^{j+1}\right). \end{array}$$

Based on the above analysis, the objective function of (13) is obviously a monotonically increasing function. At the same time, the objective function is bounded because the power of the transmitter is limited. It can guarantee algorithm convergence through the monotone convergence theorem. Algorithm 2 summarizes the proposed algorithm.

More details about specifying the value of the parameter ϵ were provided in [24], which mainly controls the accuracy and convergence of the ellipsoid sub-gradient algorithm. Moreover, both $\eta_{1}$ and $\eta_{2}$ are the convergence parameters of the proposed iterative algorithms. As the convergence parameter decreases, the number of iterations needed for the algorithm to converge increases accordingly. In other words, the accuracy of the sub-optimum values results from the iterative algorithms increase through keeping the convergence constants very small.
**Algorithm 2:** The proposed alternation optimization based method.**Input**: $H,A,\sigma^{2},C,P_{r},P_{x},\epsilon ,Z=\alpha I,\alpha \gg 1,\lambda_{1}\geq 0,\lambda_{2}\geq 0,\beta_{1}\geq 1,\beta_{2}\geq 0,\eta_{1},\eta_{2}$. $\mathrm{Initialization}:j=0,m=1,{\mathrm{SIN}R}_{r}^{0}=0,\Omega^{0}=P_{r}/NI$. **Repeat**    1. Obtain $R_{x}^{j}$ using Algorithm 1;     Repeat
      $\mathrm{Initialization}:m=1,\Omega^{j(m-1)}=\Omega^{j}$.       1. Calculate $\Phi^{jm}={\left(A\Omega^{j(m-1)}A^{H}+\sigma^{H}I\right)}^{-1}$;       2. Compute $\Omega^{jm}$ according to the given $\Phi^{jm}$ and $R_{x}^{j}$;       3. Calculate ${\mathrm{SIN}R}_{r}^{m+1}$ with $R_{x}^{j}$ and $\Omega^{jm}$
      4. m=m+1;     $\mathrm{until}\;|{\mathrm{SIN}R}_{r}^{m+1}-{\mathrm{SIN}R}_{r}^{m}|\leq \eta_{1}$.     1. $\Omega^{j}=\Omega^{jm}$;     2. Calculate ${\mathrm{SIN}R}_{r}^{j+1}$ with $R_{x}^{j}$ and $\Omega^{j}$
    3. j=j+1; $\mathrm{until}\;|{\mathrm{SIN}R}_{r}^{j+1}-{\mathrm{SIN}R}_{r}^{j}|\leq \eta_{2}$. **Output**: ${\mathrm{SINR}}_{r}$.

## 4. Simulation Results

In this section, we present numerical results to validate the merits of the proposed spectrum sharing strategy between the MIMO radar and V2V communication. The proposed work is suitable to be implemented in different vehicular communication technologies including VANET, WiFi, or IEEE 802.11ad [27]. The change was in the characteristics of the channel itself (blockage, path-loss characteristics, Doppler effect). We compared the proposed spectrum sharing strategy with other baseline approaches. In the first baseline approach, the communication system designed $R_{x}$ to minimize the transmitted power and satisfy a certain average capacity. However, it did not give any concern to the interference from the radar system, i.e., it was denoted in the results as “no P and selfish communication”. In the second baseline approach, the communication system designed $R_{x}$ to minimize the interference with the radar subject to certain communication capacity constrains, i.e., it was denoted in the result as “no P and design $R_{x}$ communication”. With loss of generality, we assumed that the coefficient of channel H was independent identically distributed zero-mean and unit-variance circularly symmetric complex Gaussian distributed, i.e., CN(0,1). We also assumed that $\epsilon =10^{-4}$. The parameters $\eta_{1},\eta_{2}$ were equal, and their value was $10^{-2}$. The number of targets was K3. The parameters of MIMO radar could be expressed as: (1)$\alpha_{k}∼\mathrm{CN}\left(0,1\right)$. (2) $\theta_{k}$ for 1≤k≤K followed a uniformly distribution within $\left[-\frac{1}{2},\frac{1}{2}\right]$. The number antennas at vehicle A and vehicle B was equal to 16.

**Figure 2:**
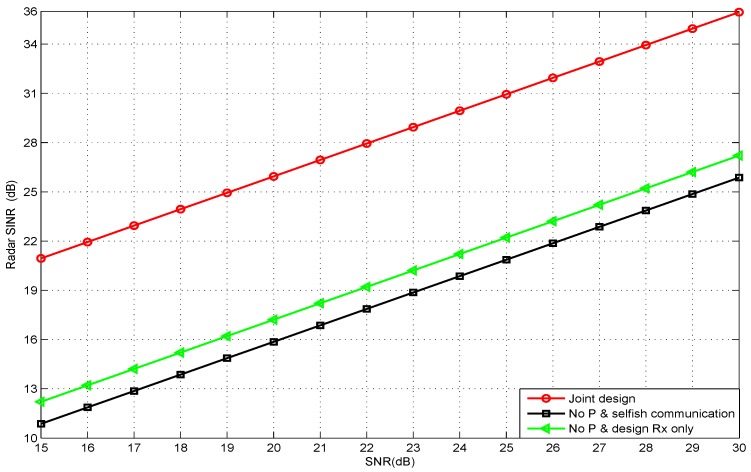
The radar SINR vs. its input SNRs under capacity threshold value 5 bits/s/Hz.

Figure 2 shows the output SINR of the automotive radar versus its input signal-to-noise ratio (SNR), when the communication capacity threshold was given a value of 5 bits/s/Hz. It was obvious that, as the SNR increased, the radar SINR increased monotonically. In addition, the SINR of “no *P* and design $R_{x}$ only” was larger than the case with “no *P* and selfish communication”. This was because the former considered how to eliminate the interference of communication to the radar. In particular, we could find that the proposed joint design method could achieve higher SINR than that of the other two methods. The reason was that the proposed method took into account how to design *P* to focus the radar power on the targets and reduce the interference to the communication receiver. At the same time, it considered how to design the $R_{x}$ to minimize the interference to the radar receiver.

**Figure 3:**
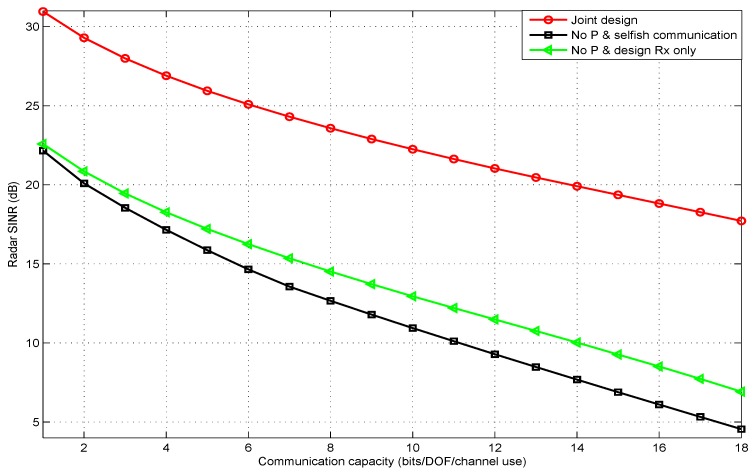
Radar SINR under different communication capacity (QoS) thresholds.

Figure 3 shows the automotive radar SINR versus QoS in terms of communication capacity threshold, at an input radar SNR equal to 20dB. We could see from the simulation result that the performance of “no Pand selfish communication” was very close to that achieved with “no P and design $R_{x}$ only”, when the communication capacity threshold equaled one (bps/Hz). The reason behind that was that vehicle A received lower power from the signal transmitted from vehicle B. Therefore, the interference power received by the radar receiver was very low. In addition, the performance of the joint design method was better compared to the other legacy baseline method, because designing the radar precoding matrix could focus the radar power on the other target.

**Figure 4:**
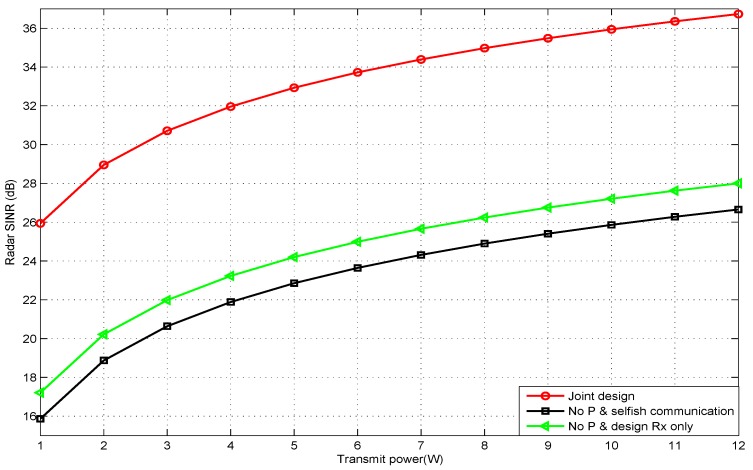
Radar SINR under different radar transmitted power budgets.

Figure 4 shows the output SINR of the radar versus different radar transmitted power budgets, at constant a communication capacity C equal to five (bps/Hz) and input SNR equal to 20 dB. It was obvious that the SINR of the automotive radar increased with the transmitted power due to the increase of the power of the echo signal. Additionally, the performance of the joint design method was better than that of the other baseline method. This was because the joint design approach improved the performance of the radar by optimizing both $R_{x}$ and Ω.

## 5. Conclusions

In this paper, we proposed a transmit beampattern based spectrum sharing scheme to achieve peaceful coexistence between automotive MIMO radar and V2V communication, i.e., finding out the optimal transmit waveform of communication and the precoding matrix of the automotive MIMO radar to maximize the SINR of the automotive radar receiver subject to the QoS in terms of the communication capacity and the transmitted power constraints. In order to solve this optimization problem, the alternating optimization approach augmented with the sub-gradient ellipsoid Lagrange dual-decomposition method was used to solve this non-convex problem. The analysis results showed that the Lagrange dual-decomposition method based on the ellipsoid method had a lower computational complexity than the conventional interior-point method. The simulation results proved that our proposed joint design scheme outperformed the baseline approaches.

## Abbreviations

The following abbreviations are used in this manuscript:

V2V	vehicle-to-vehicle
MIMO	multiple-input multiple-out
SINR	signal-to-interference plus noise ratio
5G	fifth-generation
ULA	uniform linear arrays
SVD	singular-value decomposition
INR	interference plus noise rate
DoF	degree of freedom

## Appendix A

After some mathematical transformations, the Lagrangian function of (10) can be written as:

(A1) $$\begin{array}{cc} \; & L=\mathrm{Tr}\left(\left(HH^{H}+\lambda_{2}I\right)R_{x}\right)-\lambda_{1}\log_{2}\left|I+P_{inc}^{-1}HR_{x}H^{H}\right|+\lambda_{1}C-\lambda_{2}P_{c}. \end{array}$$

Let $T=(HH^{H}+\lambda_{2}I)$ and ${\bar{R}}_{x}=T^{\frac{1}{2}}R_{x}T^{\frac{1}{2}}$; the Lagrangian function of (A1) can be described as:

(A2) $$\begin{array}{cc} \; & L=\mathrm{Tr}\left({\bar{R}}_{x}\right)-\lambda_{1}\log_{2}\left|I+P_{inc}^{-1}HT^{-\frac{1}{2}}{\bar{R}}_{x}T^{-\frac{1}{2}}H^{H}\right|+\lambda_{1}C-\lambda_{2}P_{c}. \end{array}$$

Let $\tilde{H}=P_{inc}^{-\frac{1}{2}}HT^{-\frac{1}{2}}$. Using singular-value decomposition (SVD), we have:

(A3) $$\begin{array}{c} \tilde{H}=U\Theta V^{H}, \end{array}$$

where $\Theta =\mathrm{diag}(\left[\sigma_{1},\cdots ,\sigma_{T}\right])$ with T=min(M,N).

Substituting (A3) into (A2) and after some mathematical manipulations, we obtain:

(A4) $$\begin{array}{cc} \; & L=\mathrm{Tr}\left({\bar{R}}_{x}\right)-\lambda_{1}\log_{2}\left|I+U\Theta V^{H}{\bar{R}}_{x}V\Theta^{H}U^{H}\right|+\lambda_{1}C-\lambda_{2}P_{c}. \end{array}$$

According to the characteristic of the determinant $\log_{2}\left|I+\mathrm{BC}\right|=\log_{2}\left|I+\mathrm{CB}\right|$ and ${\mathrm{UU}}^{H}=I$, we have:

(A5) $$\begin{array}{c} L=\mathrm{Tr}\left({\bar{R}}_{x}\right)-\lambda_{1}\log_{2}\left|I+\Lambda V^{H}{\bar{R}}_{x}V\right|+\lambda_{1}C-\lambda_{2}P_{c}, \end{array}$$

where $\Lambda =\Theta^{H}\Theta =\mathrm{diag}\left(\left[\sigma_{1}^{2},\cdots ,\sigma_{T}^{2}\right]\right)$. Since ${\mathrm{VV}}^{H}=I$ and Tr(ABC)=Tr(CAB), we can get:

(A6) $$\begin{array}{c} \mathrm{Tr}\left({\bar{R}}_{x}\right)=\mathrm{Tr}\left(V{\bar{R}}_{x}V^{H}\right). \end{array}$$

Let ${\tilde{R}}_{x}=V{\bar{R}}_{x}V^{H}$. Using (A6) and (A5), we have:

(A7) $$\begin{array}{c} L=\mathrm{Tr}\left({\tilde{R}}_{x}\right)-\lambda_{1}\log_{2}\left|I+\Lambda {\tilde{R}}_{x}\right|+\lambda_{1}C-\lambda_{2}P_{c}. \end{array}$$

According to the Hadamard inequality, when ${\tilde{R}}_{x}$ is the diagonal matrix, the Lagrangian function takes the maximum value. We assume that the optimal ${\tilde{R}}_{x}$ equals $\Sigma =\mathrm{diag}(\left[\vartheta_{1},\cdots ,\vartheta_{M}\right])$. After some mathematical manipulations, (A7) can be expressed as:

(A8) $$\begin{array}{c} L=\sum_{i=1}^{M}\vartheta_{i}-\lambda_{1}\sum_{i=1}^{M}\log_{2}\left(1+\sigma_{i}^{2}\vartheta_{i}\right)+\lambda_{1}C-\lambda_{2}P_{c}. \end{array}$$

Let $\frac{dL}{d\vartheta_{i}}=1-\frac{\lambda_{1}\sigma_{i}^{2}}{(1+\sigma_{i}^{2}\vartheta_{i})\ln2}$. Since $\frac{dL}{d\vartheta_{i}}=0$ and the power of the transmitter must be greater than zero, we have:

(A9) $$\begin{array}{c} \vartheta_{i}={\left(\frac{\lambda_{1}}{\ln2}-\frac{1}{\sigma_{i}^{2}}\right)}^{+}, \end{array}$$

where ${\left(\right)}^{+}=\max\left(0,.\right)$ . After the same mathematical manipulations, we can obtain the other $\vartheta_{i},i\in \left\{1,\cdots ,M\right\}$. Based on the above manipulations, we have:

(A10) $$\begin{array}{c} {\bar{R}}_{x}=V\Sigma V^{H}. \end{array}$$

Since ${\bar{R}}_{x}=T^{\frac{1}{2}}R_{x}T^{\frac{1}{2}}$, the optimal $R_{x}^{*}$ can be expressed as:

(A11) $$\begin{array}{c} R_{x}^{*}=T^{-\frac{1}{2}}V\Sigma V^{H}T^{-\frac{1}{2}}. \end{array}$$

## Appendix B

Appendix B gives a specific derivation of (20)

(A12) $$\begin{array}{c} \log_{2}\left|I+P_{\mathrm{inc}}^{-1}HR_{x}H^{H}\right|\\ \overset{1}{=}\log_{2}|P_{\mathrm{inc}}+HR_{x}H^{H}|+\log_{2}\left|P_{\mathrm{inc}}^{-1}\right|\\ \overset{2}{=}\log_{2}|A\Omega A^{H}+\sigma^{H}I+HR_{x}H^{H}|+\max_{\Phi }-\mathrm{Tr}\left(\left(A\Omega A^{H}+\sigma^{H}I\right)\Phi \right)+\log_{2}\left|\Phi \right|+N\\ \overset{3}{=}\log_{2}\left(\right|G^{-1}A\Omega A^{H}+I|)+\log_{2}\left(|G\Phi |\right)-\mathrm{Tr}\left(A^{H}\Phi A\Omega \right)-\mathrm{Tr}\left(\sigma^{2}\Phi \right)+N. \end{array}$$

The equations $\overset{1}{=},\overset{2}{=}$ are established because of $\log_{2}\left|\mathrm{BC}\right|=\log_{2}\left|B\right|+\log_{2}\left|C\right|$ and Lemma 1.
